# LRF Promotes Indirectly Advantageous Chromatin Conformation via *BGLT3*-lncRNA Expression and Switch from Fetal to Adult Hemoglobin

**DOI:** 10.3390/ijms23137025

**Published:** 2022-06-24

**Authors:** Vasiliki Chondrou, Athanasios-Nasir Shaukat, Georgios Psarias, Katerina Athanasopoulou, Evanthia Iliopoulou, Ariadne Damanaki, Constantinos Stathopoulos, Argyro Sgourou

**Affiliations:** 1 Biology Laboratory, School of Science and Technology, Hellenic Open University, 26335 Patras, Greece; geo.psarias@gmail.com (G.P.); athanasopoulou.aikaterini@ac.eap.gr (K.A.); evanthia390@gmail.com (E.I.); ar.damanaki@gmail.com (A.D.); 2 Department of Biochemistry, School of Medicine, University of Patras, 26504 Patras, Greece; a.sokat@upnet.gr (A.-N.S.); cstath@upatras.gr (C.S.)

**Keywords:** hemoglobin switch, *BGLT3*-lncRNA expression, chromatin conformation, LRF/*ZBTB7A* overexpression

## Abstract

The hemoglobin switch from fetal (HbF) to adult (HbA) has been studied intensively as an essential model for gene expression regulation, but also as a beneficial therapeutic approach for β-hemoglobinopathies, towards the objective of reactivating HbF. The transcription factor LRF (Leukemia/lymphoma-related), encoded from the *ZBTB7A* gene has been implicated in fetal hemoglobin silencing, though has a wide range of functions that have not been fully clarified. We thus established the LRF/*ZBTB7A*-overexpressing and *ZBTB7A*-knockdown K562 (human erythroleukemia cell line) clones to assess fetal vs. adult hemoglobin production pre- and post-induction. Transgenic K562 clones were further developed and studied under the influence of epigenetic chromatin regulators, such as DNA methyl transferase 3 (DNMT3) and Histone Deacetylase 1 (HDAC1), to evaluate LRF’s potential disturbance upon the aberrant epigenetic background and provide valuable information of the preferable epigenetic frame, in which LRF unfolds its action on the β-type globin’s expression. The ChIP-seq analysis demonstrated that LRF binds to γ-globin genes (*HBG2/1*) and apparently associates BCL11A for their silencing, but also during erythropoiesis induction, LRF binds the *BGLT3* gene, promoting *BGLT3*-lncRNA production through the γ-δ intergenic region of β-type globin’s locus, triggering the transcriptional events from γ- to β-globin switch. Our findings are supported by an up-to-date looping model, which highlights chromatin alterations during erythropoiesis at late stages of gestation, to establish an “open” chromatin conformation across the γ-δ intergenic region and accomplish β-globin expression and hemoglobin switch.

## 1. Introduction

Perturbations in production and operation of the human β-type globin genes (“*HBB* locus”) are designated as β-hemoglobinopathies and are mainly inherited by the autosomal recessive pattern. Of the most common genetic disorders among the human population, related to the “*HBB* locus”, are β-thalassemias and Sickle Cell Anemia (SCA). Clinical practice has shown that despite identical disease-causing mutations, the pathological phenotype of disease may vary significantly between individuals. This is partly due to genetic variants of the “*HBB* locus” including the remote regulatory region (LCR-Locus Control Region) [1] and epigenetic factors affecting chromatin structure [2,3,4]. DNA methylation, histone acetylation, intragenomic lncRNA expression and post-transcriptional attack of several β-type globin genes’ modifier factors by miRNAs, are of the most important mechanisms involved in epigenetic regulation of the “*HBB* locus” [5,6,7,8,9].

Therapeutic schemes aiming to reactivate fetal hemoglobin (HbF) based in pharmacological factors’ administration have been applied to the clinic for the treatment of β-hemoglobinopathies with dubious results, because of the moderate and heterogeneous HbF expression levels produced, the lack of specificity, and the observed cytotoxicity and mutagenicity in the long run [10,11]. Beneficial contribution of high HbF production to the severe clinical symptoms of β-hemoglobinopathies has been observed at the Hereditary Persistence of HbF in adults (HPFH syndrome), exhibiting an asymptomatic phenotype [12] and thus, the effective reactivation of γ-globin gene’s expression has been constantly at the forefront of research attempts. Clarification of the hemoglobin switch process, from fetal (HbF) to adult (HbA), is considered as a prerequisite for the successful outcome of such efforts. Even though hemoglobin switching is under intensive study, there are still some “dark”, mainly epigenetic, regulatory rules awaiting to be enlightened.

The leukemia/lymphoma-related (LRF/*ZBTB7A*) transcription factor has been characterized as a critical key regulator of HbF in erythroid cells, acting independently from the main repressor, BCL11A (B Cell CLL/Lymphoma 11A) [7,13,14], and exerting its repressive activity through recruitment of the NuRD (Nucleosome Remodeling) complex. Interaction with the NuRD complex regulates chromatin by reducing activating histone marks and inhibits fetal globin genes’ transcription, indicating LRF as an indirect epigenetic component conferring its repressing activity at the late stages of erythrocytes maturation, during hemoglobin switch from HbF to HbA. LRF deficient human CD34+ hematopoietic stem and progenitor cells (HSPC) show a delay in erythroid differentiation, whereas double LRF/*ZBTB7A*-knockout HUDEP-2 cells (human immortalized erythroid line, which requires doxycycline removal to achieve terminal differentiation) exhibit a significantly high γ/β globin ratio [7].

The present study aimed to determine the influence of LRF to the balance between γ/β-globin production at both LRF-overexpression or LRF-knockout conditions. We thus established stably overexpressing (OE) LRF and LRF-knockdown (KD) K562 human erythroleukemic clones, which were studied before and during response to hemoglobin induction with chemical treatment [15]. K562 cells have been extensively used as an in vitro model for the study of molecular mechanisms underlying human globin genes’ regulation [16] and constitutively express HbF; although, the cell line originates from an adult, rendering this genetic/epigenetic background the appropriate frame for attainment and study of the switch process from γ- to β-globin expression. In a parallel experiment, K562 clones were stably modified to display an aberrant epigenetic landscape, with the objective to uncover potential epigenetic issues related to globin’s expression regulation across the “*HBB* locus”.

## 2. Results

### 2.1. LRF/ZBTB7A Overexpression in Transfected K562 Clones and Hemin/EPO Treatment Induces Preferentially β- Than γ-Globin Expression

**Figure 1:**
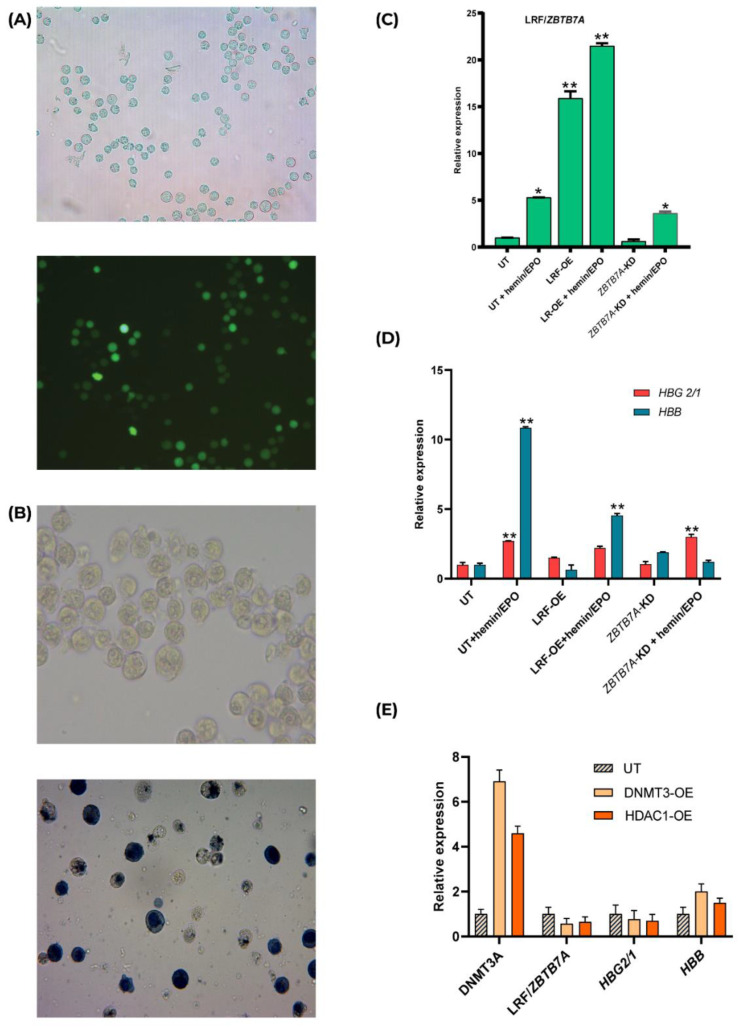
LRF-, DNMT3A-, HDAC1-overexpressing (OE) K562 clones and induction of erythropoiesis. (**A**) eGFP detection in LRF-overexpressing (OE) K562 cells, 2 months post transfection. Images derived from phase contrast (up) and fluorescence (down) microscopy. (**B**) Benzidine staining of untreated (up) and treated with 30 μΜ hemin/5 ng/mL ΕPOK562 cells. Hemoglobinized cells were stained dark blue. (**C**) Relative expression of LRF/*ZBTB7A* in LRF-overexpressing (OE) and *ZBTB7A*-knockdown (KD) K562 clones, pre- and post-hemin/EPO induction. (**D**) Relative expression of *HBG2/1* and *HBB* in LRF-OE and *ZBTB7A*-KD K562 clones, pre- and post-hemin/EPO induction. (**E**) Relative expression of *DNMT3A*, *HDAC1*, LRF/*ZBTB7A*, *HBG2/1* and *HBB* in DNMT3A-OE and HDAC1-OE K562 clones respectively. Values are normalized to untransfected (UT), non-induced cells. * *p* < 0.05, ** *p* < 0.01.

We have developed an in vitro study system composed of human transgenic K562 cell clones, either overexpressing or diminishing LRF/*ZBTB7A* expression, towards the objective of unraveling the function of the transcription factor LRF, encoded from the *ZBTB7A* gene, known to act during hemoglobin transversion by inhibiting γ-globin (*HBG2/1*) genes’ expression [7,14]. LRF-overexpression was efficiently established by the episomal vector LRF/*ZBTB7A*-GFP for longer than 60 generations during continuous culture of K562 cells in the presence of (400 μg/mL) G418 antibiotic (Figure 1A). *ZBTB7A* knockdown (KD) K562 clones were obtained by a CRISPR/Cas9 system guided to target the first protein coding exon of *ZBTB7A,* with sequence specific gRNAs (Figure 5C). Recombination between donor DNA carrying the Puromycin-GFP cassette and the first exon of the *ZBTB7A* sequence led to genetically engineered K562 cell clones surviving in the presence of 6 μg/mL Puromycin. Sequencing analysis downstream and upstream from predicted insertion sites revealed heterozygous knockout K562 clones and the insertion of the Puromycin-GFP cassette in exogenous regions as well, which were anticipated due to the high genetic instability nature of the K562 karyotype [17]. LRF expression was quantified and was found to be accelerated by 15-fold in LRF-overexpressing (OE) clones, whereas it was reduced to half expression in ZBTB7A- knockdown (KD) K562 clones (Figure 1C).

Untransfected and transfected either long-term LRF-OE or *ZBTB7A*-KD K562 clones, were subsequently treated with hemin/erythropoietin (EPO) to urge cell differentiation and hemoglobin production. K562 cell line behaves like undifferentiated early pluripotent hematopoietic progenitors and upon chemical induction with hemin/EPO differentiate towards an erythroid character [18]. Furthermore, another major characteristic of K562 cell line is that although it derives from an adult patient and a BCL11A XL transcript is generated, fetal (HbF) hemoglobin (*HBG2/1*) expression still dominates, whereas adult (HbA) hemoglobin production (*HBB* expression) is totally suppressed [19].

Hemin/EPO cocktail induced total hemoglobin expression up to 48% during a 3-h interval (Figure 1B). In particular, *HBG2/1* expression was increased by at most 2.7-fold in untransfected and 2.2-fold in LRF-overexpressing K562 clones. *HBB* expression was elevated at much higher levels. by 10.9-fold in untransfected and 4.5-fold in LRF-overexpressing K562 clones (*p* < 0.01). Contrariwise, *ZBTB7A*-KD K562 clones upon hemin/EPO treatment showed 3-fold elevation in *HBG2/1* and an insignificant change in *HBB* expression levels (Figure 1D), confirming results from previously conducted research [7,13,14].

**Figure 2:**
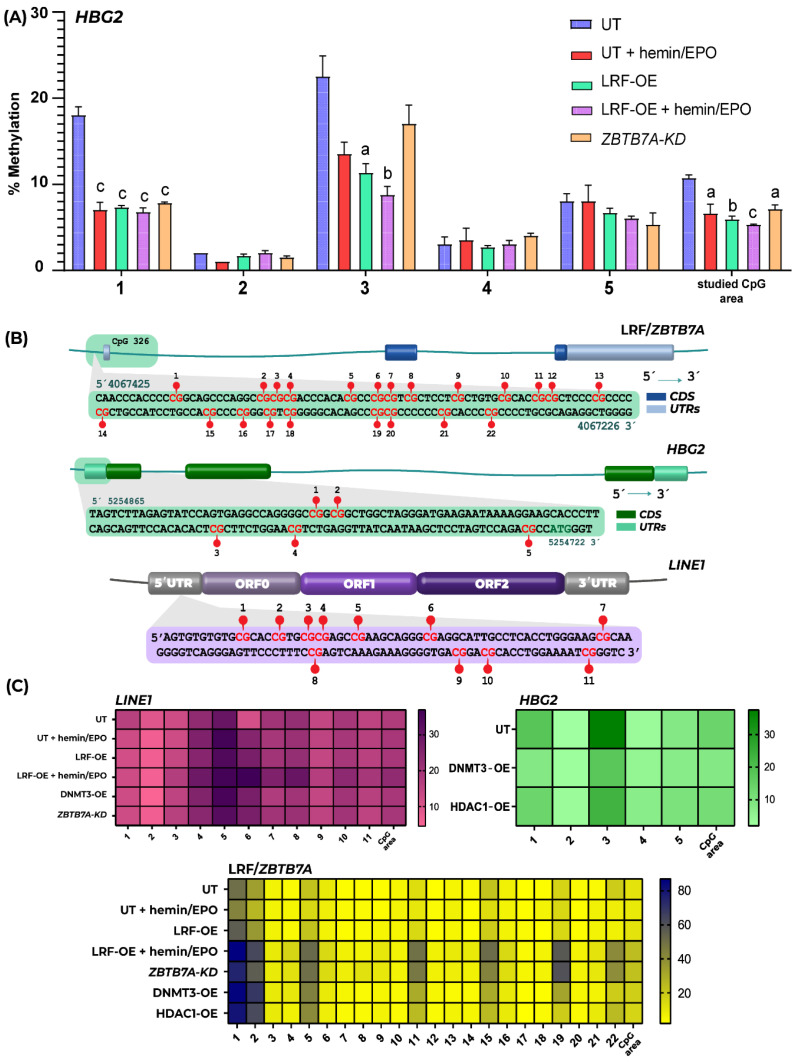
Methylation pattern of CpGs at *HBG2*, LRF/*ZBTB7A* and *LINE1* genetic loci. (**A**) The methylation levels of specific CpG sites lying at the 5′ prime end of *HBG2* gene. Data are presented as mean ± SE, a indicates *p* < 0.05, b indicates *p* < 0.01 and c indicates *p* < 0.001. (**B**) Schematic representation of *HBG2* and LRF/*ZBTB7A* gene loci and the *LINE1* transposable element. The detailed genomic positions and CpG dinucleotides analyzed are presented. (**C**) Heatmap visualization of the Cytosines’ methylation levels of CpG sites within *HBG2* and *ZBTB7A* promoter regions and 5′UTR of *LINE1*. Methylation status of the whole CpG area in each locus is also depicted. Each square corresponds to one CpG site analyzed; data are presented as mean percentage methylation.

Further to qPCRs, the methylation status of CpGs lying at the promoter region of *HBG2*, which have been shown to be hypermethylated in adult erythroblasts, as an additional mechanism leading to “closed” chromatin conformation required for γ-globin silencing [20], was estimated with Pyrosequencing. Methylation of Cytosines followed by Guanosines located at *HBG2* promoter region, more specifically CpG1, CpG3 and CpG6, showed significantly lower values of methylation in LRF-overexpressing K562 clones, further depleted in both untransfected and LRF-overexpressing K562 clones after addition of hemin/EPO (Figure 2A).

Data interpretation clearly manifests a favorable expression of *HBB* during hemin/EPO treatment of untransfected and LRF-OE K562 cells and a minor elevation of *HBG2/1* expression, although the drastic hypomethylation of the *HBG2* promoter would potentially hyper boost gene’s transcription levels. *ZBTB7A*-KD K562 clones fail to increase *HBB* expression during hemoglobin induction, though *HBG2/1* were significantly upregulated (Figure 1D).

### 2.2. Epigenetic Events Promoted by Overexpression of DNMT3A and HDAC1 Leads to Insubstantial Changes in γ- and β-Globin Expression Levels

In an effort to evaluate LRF/*ΖΒTB7A* as an epigenetic reader we have produced transgenic K562 clones either expressing the core enzymatic center of the de novo acting DNA methyl-transferase DNMT3A or the ubiquity-expressed Histone Deacetylase HDAC1 and double expressing clones with DNMT3A and LRF/*ΖΒTB7A*. In mammals, DNA methyltransferase enzymes comprise of two parts: a large multidomain N-terminal part of variable size, which has regulatory functions, and a C terminal catalytic part. The N-terminal part of DNMT3A is followed by a proline-tryptophan-tryptophan-proline (PWWP) domain, an ATRX-DNMT3-DNMT3L-type zinc finger (ADD) domain, whereas the smaller C-terminal part, is common for all DNMTs and harbors the active center of the enzyme, which contains six highly conserved cytosine C5-DNA methyltransferase motifs [21,22]. Due to length restrictions and stability issues designated for the DNA sequence synthesis, we have constructed and cloned within the episomal vector only the C-terminal domain acquiring methyltransferase activity of DNMT3A. However, by this option, exogenous DNMT3A overexpression reflects the action of other DNA methyltransferases too, which share the same enzymatic active center and are responsible to perform de novo DNA methylation. HDAC1, the other epigenetic remodeler, removes acetyl-groups from the histone tails leading to more condensed chromatin conformation.

Transfected K562 clones were constantly cultivated for 2 months and studied under the prevailing frame of epigenetic instability, stimulated by the elevated expression levels of DNMT3 and HDAC1. DNMT3A and HDAC1 were upregulated by 6.9- and 4.6-fold respectively (Figure 1E). DNMT3A/LRF double transfected K562 clones unexpectedly overexpressed LRF but not DNMT3A, suggesting a possible overlap of the LRF function, covering DNMT3A. Thus, results derived from double-transfected clones will not be further discussed. DNA methylation profiles of genetic loci were assessed to confirm epigenetic disturbance and *HBG2/1* and *HBB* expression were estimated in an attempt to study the hemoglobin expression profile during aberrant methylation across the genome.

In previous studies, *ZBTB7A* CpG island 326 has been indicated as a delicate sensor of DNA methylation and associated with the impaired pharmacological induction of HbF upon hydroxyurea treatment of β-hemoglobinopathies patients [13]. Additionally, repetitive sequences such as the Long Interspersed Nuclear Element-1 (LINE-1) repeats, are normally hypermethylated across the human genome to suppress their transcription and retrotransposition. LINE-1 represents almost 18% of the genome sequences and its methylation status has been suggested as a surrogate marker for estimating global DNA methylation [23]. Both genetic areas were analyzed in transfected K562 clones for divergent methylation profiles, utilizing targeted Pyrosequencing analysis. LINE-1 CpGs were hypomethylated in all experimental conditions tested, as in untransfected K562 cells (Figure 2B,C), indicating up-regulation of expression and capability of transposition throughout the genome, contributing to genomic instability, one of the hallmarks of cancer in general.

CpG 326 of *ZBTB7A* was significantly hypermethylated at positions CpG1, CpG2, CpG5, CpG11, CpG15, CpG19 and CpG22 (Figure 2B,C), under the influence of both epigenetic modifiers’ overexpression, either DNMT3A-OE or HDAC1-OE. *HBG2* proximal promoter methylation levels were also evaluated and found unaltered compared to untransfected cells (Figure 2B). Epigenetic changes studied in transfected K562 genome were equivalent at both DNMT3A-OE and HDAC1-OE status.

Performed qPCRs revealed that *HBG2/1*, *HBB* and LRF/*ZBTB7A* expression levels were unaffected (Figure 1E). Results lead to the conclusion that the K562 genome hypermethylation in DNMT3-OE and HDAC1-OE clones was partially and selectively achieved, nevertheless there was not any remarkable impact observed on LRF/*ZBTB7A*, *HBG2/1* and *HBB* expression, implying that epigenetic imbalance does not influence LRF’s function.

### 2.3. ChIP-Seq Analysis Reveals a Novel Function of LRF’s Regulatory Properties on *BGLT3* Gene

To identify enriched loci with recognition binding sites for LRF within the K562 genome, the chromatin immunoprecipitation method with antibodies against LRF followed by the next generation sequencing of precipitated DNA fragments, was utilized. Untransfected and LRF-OE transfected clones were subjected to analysis pre- and post-erythropoiesis induction with hemin/EPO (four experimental conditions). To confirm the reliability of our results we have downloaded and compared a relevant ChIP-seq dataset from the Sequence Read Archive (SRA) (Accession number SRR6006200). To identify differentially LRF-enriched regions between experimental conditions and correlate our results to corresponding ChIP-seq data created by other research groups, read normalization was conducted to mitigate technical variance.

**Figure 3:**
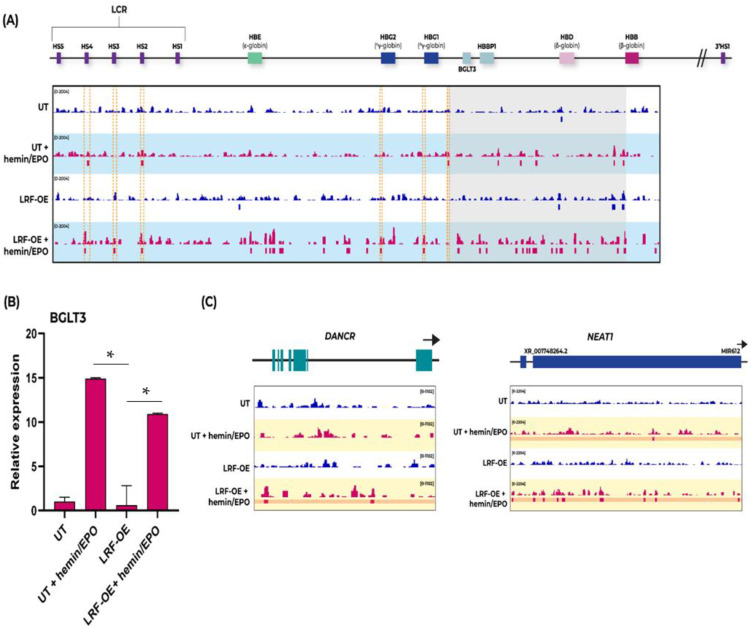
LRF/*ZBTB7A* occupancy sites at “*HBB* locus” and *BGLT3*-lncRNA expression levels. (**A**) Tracks display ChIP-seq data of LRF/*ZBTB7A* occupancy and calculated peak enrichment sites for each sample. Reads for untransfected (UT) and LRF-overexpressing (OE) K562 cells, before and after erythropoiesis induction across “*HBB* locus” including βLCR are depicted. LRF/*ZBTB7A* occupancy sites starting from *BGLT3* gene and across the intergenic region towards the *HBB* gene are highlighted in the grey background. (**B**) Relative expression of *BGLT3*-lnc RNA in LRF-overexpressing (OE) K562 cells pre- and post-hemin/EPO induction. Values were normalized to untransfected (UT) non induced cells. * *p* < 0.05. (**C**) ChIP-seq data of LRF/*ZBTB7A* occupancy sites pre- and post-hemin/EPO induction at genetic loci expressing DANCR- and NEAT1-lncRNAs, reported to be involved in erythropoiesis.

LRF was shown to be broadly distributed across the genome with a high rate of enriched regions, further increased upon erythropoiesis (hemin/EPO induction) (Figure 3A). Lengthwise, “*HBB* locus” LRF occupied fetal (*HBG2/1*) and *BGLT3* genes with significant enrichment at post-erythropoiesis condition in untransfected and more evident in LRF-overexpressing K562 clones. Across βLCR, LRF signals were enriched at HS2 and HS4 (DNAse hypersensitive sites) in untransfected and at HS2, HS3 and HS4 in LRF-OE K562 cells, both during hemin/EPO induction. LRF-OE pre-hemin/EPO induction failed to highlight any LRF’s connection to βLCR. LRF-ChIP binding signals were extended to the adult *HBB* gene at post-erythropoiesis in untransfected and in both LRF-overexpressing clones (pre- and post-hemin/EPO induction). Observed LRF occupancy sites at *HBG2/1* were adjacent to BCL11A [24], which can be considered as critical and necessary for the co-silencing of *HBG2/1* genes. Moreover, LRF can potentially master *BGLT3* gene’s up-regulation and as a consequence the enhancement of *HBB* expression in adults.

To evaluate *BGLT3* expression during experimental conditions studied, we carried out qPCRs with sequence specific primer sets. *BGLT3*-lncRNA expression was evident only in hemin/EPO induced K562 clones (Figure 3B) at comparable values; 14.9-fold elevation in untransfected and 10.9-fold elevation in LRF-overexpressing clones, but not in LRF-OE pre-hemin/EPO induction, indicating that extra erythroid specific transcription factors’ participation and specific βLCR HSs activation are required for co-operation and full manifestation of this phenomenon. K562 cell erythroid differentiation after chemical induction is regulated by the Raf/MEK/ERK pathway and the transcription factor ETV6, a member of the transcription factor E-Twenty Six (ETS) family [25]. Both hemin and erythropoietin, administered to K562 cells in the present study, induce a rapid activation of the ERK pathway and hemoglobin production [26]. The profiling of hemin induced proteins in K562 has revealed a wide range of protein molecules involved in diverse activities, such regulators of intracellular trafficking, chaperones, modulators of mitochondrial multi-electron transfer in the respiratory chain, gene expression regulators, and members along signal transduction pathways etc. [27]. Nevertheless, the exogenous *ZBTB7A* gene lacks all upstream and downstream genomic regulatory regions responsible for cell signaling response, and as a consequence, the hemin/EPO induction process has no apparent effect on it. This is consistent with results of the study where comparable results from untransfected and LRF-overexpressing K562 clones post-hemin/EPO induction, were obtained.

## 3. Discussion

The present study attempted to highlight epigenetic issues related to the hemoglobin switch from fetal (HbF) to adult (HbA), and mainly the role of LRF/*ZBTB7A* as a potential epigenetic regulator of this process. According to the ChIP-seq analysis, LRF/*ZBTB7A* binds *HBG2/1* promoter regions and immediately upstream of *BGLT3* gene in K562 cells, mainly during hemin/EPO induction. LRF/*ZBTB7A* residence is upgraded in LRF-overexpressing and untransfected K562 cells post-hemin/EPO induction, the same as *BGLT3*-lncRNA expression (Figure 3A,B). Eventually, *BGLT3*-lncRNA expression and enhancement of specific DNAse hypersensitive sites (HSs) within LCR further stimulate the epigenetic events responsible for the activation of the downstream *HBB* promoter (Figure 1D). The consequent preferential HbA hemoglobin increase is apparently co-assisted by LRF and other structural and transcription partners, also induced by hemin/EPO treatment.

**Figure 4:**
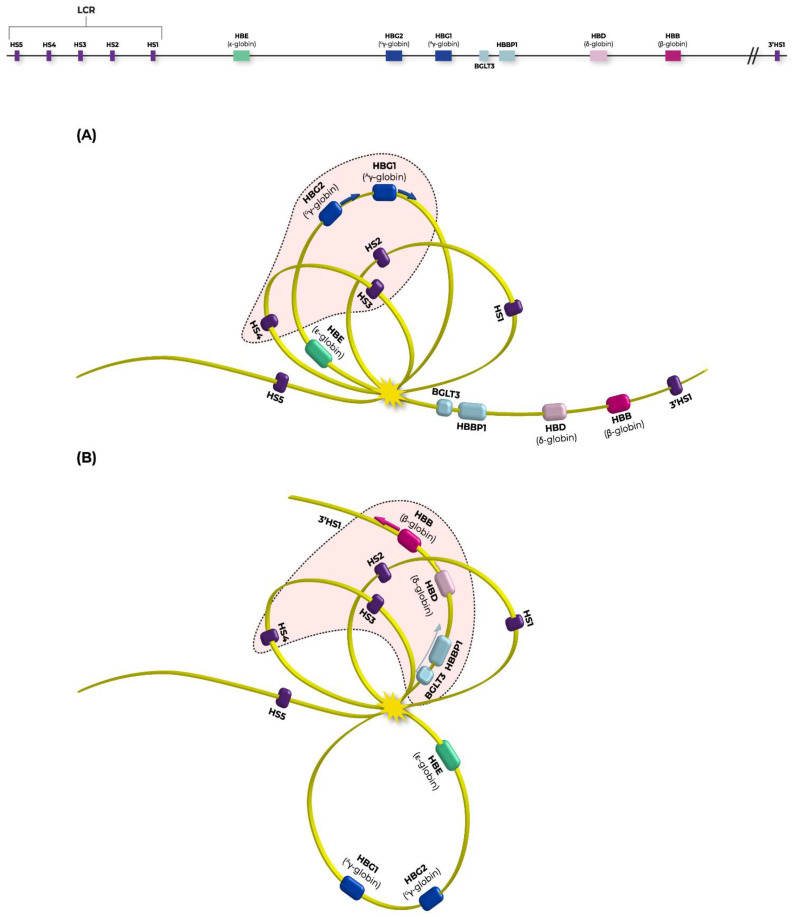
Updated looping model illustrating fetal (HbF) to adult hemoglobin (HbA) switch. (**A**) At fetal stage DNA loops promote the proximity of *HBG2* and *HBG1* (γ-globin genes) to DNAse Hypersensitive Sites (HS2, HS3 and HS4) favoring HbF expression. (**B**) During HbF to HbA transversion BCL11A and LRF (not shown for satisfying model’s simplicity) bind *HBG2/1* promoter regions, leading to their full silencing. Simultaneously, LRF promotes *BGLT3*-lncRNA expression, further releasing downstream chromatin conformation, thus supporting *HBB* expression (for detailed analyses refer to discussion). Τhe chromatin loop containing both fetal genes (*HBG2* and *HBG1*) interacting with HS2, HS3 and HS4 LCR enhancer sites, has been now replaced with the “adult”-genes’ loop including *BGLT3*, *HBBP1*, *HBD* and *HBB* genes. Chromatin modulation stimulates HbA expression at the adult stage. Arrows derived from genes indicate transcription. Yellow star represents the anchor gathering S/MAR elements of “*HBB* locus”.

During the last weeks of gestation, fetal hemoglobin (HbF) is replaced by adult (HbA), a process known to be orchestrated by the BCL11A repressor function to *HBG2/1* genes’ promoters. Apart from BCL11A, the γ-δ putative intergenic region upstream from *HBD*, encompassing the *BGLT3* gene, has been strongly implicated to *HBG2/1* silencing. This intergenic region is transcriptionally silent during the fetal stage and is also associated with high HbF levels (HPFH syndrome) in naturally occurring deletions detected in β-hemoglobinopathies patients, implying a strong potential of *BGLT3*-lncRNA expression to *HBB* induction [5,28]. Additionally, *BGLT3*-lncRNA production is induced by imatinib treatment in Bcr-Abl (translocation between chromosome 9 and 22, resulting in hybrid bcr-abl gene) positive K562 erythroleukemic cells and leads to apoptosis. Study by Guo et al., 2018, concluded that Bcr-Abl represents a *BGLT3*-lncRNA repressor probably acting through DNA hypermethylation of the region, leading to transcriptional silencing [29]. However, this notion was not proved by our methylation assays in overexpressing DNMT3A and HDAC1 K562 clones. *BGLT3*-lncRNA transcript elevation during hemoglobin switch reflects several other epigenetic modifications, additional to the already known pleiotropic activities of lncRNAs, such as chromatin remodeling, protein guidance to genomic loci via RNA-DNA interactions, transcription and RNA splicing [30,31,32]. We thus propose that during hemoglobin transversion from HbF to HbA, binding of BCL11A to *HBG2/1* is a perquisite for HbF repression, but to accomplish full repress of *HBG2/1* genes, LRF/*ZBTB7A* occupancy is required, too. Simultaneous binding of LRF at *HBG2/1* and *BGLT3* advances BCL11A’s suppression efficiency to *HBG2/1* genes and the accessible conformation of downstream chromatin up to *HBB* by *BGLT3*-lncRNA expression enhancement (Figure 4). Results from qPCRs also manifested that *HBB* expression is advanced under the influence of high *BGLT3*-lncRNA expression, in an inverse manner rather than *HBG2/1* expression (Figure 1D), despite *HBG2’s* hypomethylated status (Figure 2A), suggested as contributing to its transcriptional elevation.

Obstructed KLF1 function has been also associated with reduced levels of the γ-globin repressors, BCL11A and LRF/*ZBTB7A* and a reversal of the fetal globin genes’ expression [33,34,35]. Within the promoter region of *ZBTB7A,* are the KLF1 and GATA-1 binding sites, driving a favorable generation of a longer LRF/*ZBTB7A* transcript, comprising a 5′ prime-end extension and exclusively expressed during late erythroid differentiation [35]. However, since the exogenous *ZBTB7A* gene lacks KLF-1 and GATA-1 binding capacity, the enhancement upon hemin/EPO treatment derives only from the endogenous *ZBTB7A* gene, which explains the analogous imprinted results from LRF-overexpressing and untransfected K562.

So far, active β-type globin promoters are suggested to form 4D chromatin configurations, such as loops with βLCR tissue-specific enhancer and engagement of transcription factors, accomplishing operative networks to promote spatiotemporal transcriptional programs during distinct developmental stages. Genes in general tend to restrict their contacts with enhancers within the same TAD (Topologically Associating Domains) [36], whereas CTCF (CCCTC-binding factor) [37] has been considered as a mediator of embedding chromosomal loops in the framework of TADs and/or insulators, setting a barrier between active genes. CTCF sites are enriched in TADs’ borders, further supporting this role [38]. Inter-TADs are discrete domains within a TAD, potentially formed by chromatin S/MARs (Scaffold/Matrix Attachment Regions), which lack sequence conservation, but are regarded as anchor sequences of chromosomes to the nuclear matrix [39,40]. S/MARs are signatured by AT- or TG- rich sequences and curved DNA, with a median length ≤2 kb, usually possessing origin of replication (OriC) features. The median length of the formed chromatin loops by S/MARs is ≤31 kb [41,42,43], a considerably smaller scale than TADs, particularly of the *HBB* locus, which is composed of almost 300 kb [44]. Lengthwise, within the human *HBB* locus, four MAR elements are predicted [45] and suggested to cooperate at several developmental stages of the fetus by recruiting in a preset order: *HBE, HBG2* and *HBG1* and finally *HBB* promoters to LCR, leading to a dominant expression of a different hemoglobin at each stage. Looping models between LCR and globin genes’ promoters have long been proposed and further expanded under new enlightened data derived from current techniques [24,46].

The proposed looping model derived from the present study (Figure 4) is in consistency with recently published models and combines all basic factors influencing HbF transversion to HbA [24,43]. Additionally, this clarifies the characterization of LRF/*ΖΒΤΒ7A* as an indirect epigenetic modifier factor with an evolving role in lncRNAs’ regulation of expression. Further evidence for LRF’s regulatory properties on lncRNAs is established from its designated occupancy sites at 5′ prime-ends of *DANCR* and *NEAT1* lncRNA genes, suggested to have an involvement in erythropoiesis [47,48], which is obtained from the ChIP-seq analyses (Figure 3C).

Conclusively, it is apparent that LRF/*ZBTB7A* has an emerging role as an indirect chromatin modulator during the HbF to HbA switch. The “*HBB* locus”, including the tissue-specific LCR enhancer, is highly enriched with LRF/*ZBTB7A* recognition sites, which vary in potency during erythropoiesis and are not affected by variations in genome methylation levels. LRF certainly binds and potentially co-silences *HBG2/1* with the BCL11A transcription factor, but also enables *BGLT3*-lncRNA and apparently other erythroid specific lncRNAs’ expression, triggering HbF to HbA transversion in the adult stage of erythropoiesis.

## 4. Materials and Methods

### 4.1. Cell Culture

Human K562 erythroleukemia cells were cultured in Dulbecco’s Modified Eagle Medium (DMEM, Sigma), supplemented with 10% fetal bovine serum (FBS, Gibco—Thermo Fisher Scientific Inc., Grand Island, NE, USA), 100 units/mL penicillin and 100 μg/mL streptomycin (Sigma -Aldrich Pty Ltd, An affiliate of Merck KGaA, Darmstadt, Germany) in a humidified atmosphere at 37 °C and 5% CO_2_. They were passaged every 2 days. Erythroid differentiation was induced by treatment with 30 μΜ hemin (AppliChem GmbH, Darmstadt, Germany) and 5 ng/mL erythropoietin (EPO) (Cell signaling) for 3 h at 37 °C and 5% CO_2_ [49,50]. Cell viability was evaluated using 0.4% trypan blue stain (Fluorochem, Hadfield, UK). Total hemoglobin pre- and post-induction was obtained by Benzidine staining [51]. K562 transfections were carried out with Lipofectamine^®^ 3000 (Invitrogen- Thermo Fisher Scientific Inc., Grand Island, NE, USA) according to manufacturer’s protocol, and transgenic clones were selected by 800 μg/mL of Geneticin (G418) (Gibco—Thermo Fisher Scientific Inc., Grand Island, NE, USA) or 6 μg/mL puromycin for CRISPR/Cas9. Long-term expression of the reporter gene eGFP in transfected cells was detected by fluorescence microscopy (LEICA Microsystems, Heerbrugg, Switzerland), confirming exogenous genes’ sustainable existence.

### 4.2. Plasmid Construction

**Figure 5:**
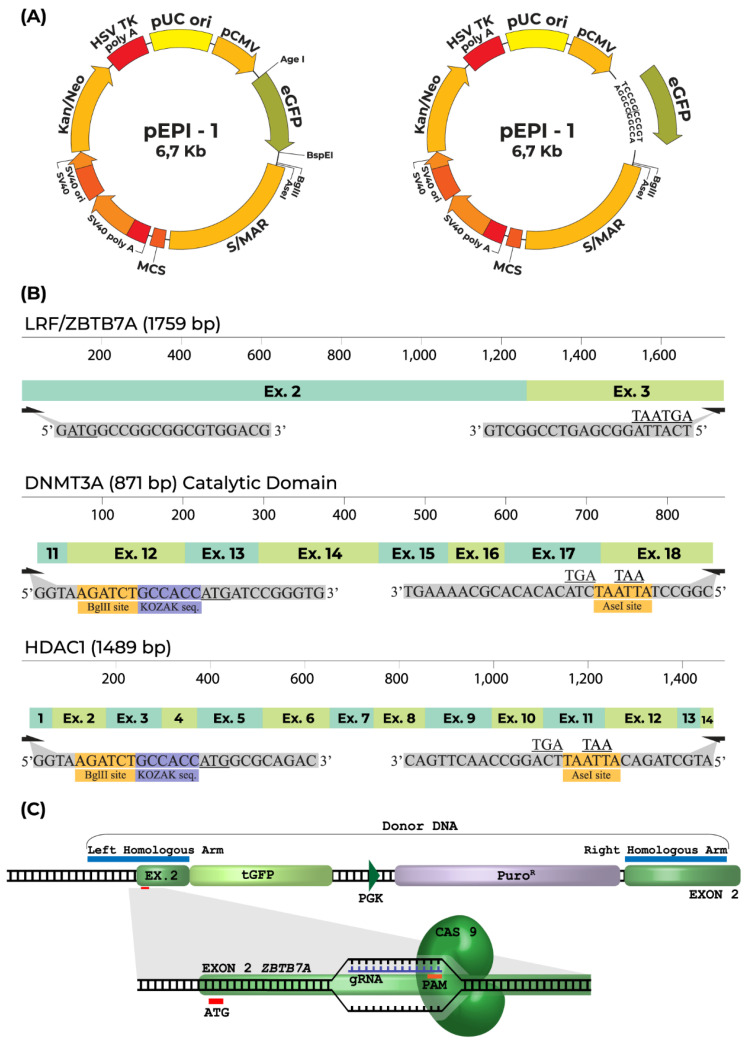
Construction of LRF/*ZBTB7A*, DNMT3A, HDAC1 episomal vectors and the LRF/*ZBTB7A* knockout CRSPR/Cas9 system. (**A**) Restriction sites for LRF/*ZBTB7A*, *DNMT3A*, *HDAC1* insertion into the episomal vector PEPI-1. Reporter gene’s eGFP sequence, between AgeI/BspEI sites, was removed. Arrows indicate the location as well as the orientation of the eGFP and the vector’s functional elements. (**B**) Schematic representation of LRF/*ZBTB7A*, catalytic domain of *DNMT3* and *HDAC1* sequences incorporated within PEPI-1. Kozak sequence and restriction endonuclease sites added (in PCR primers) are marked with orange and blue color respectively. Start and stop codons are underlined. (**C**) Schematic illustration of CRISPR/Cas9 mediated LRF/*ZBTB7A* gene knockout through homology-directed repair mechanism. CRISPR/Cas9 cuts the double stranded DNA after first codon (ATG) indicated as a red rectangle. Position of left and right homology arm is indicated with blue rectangle. Edited *ZBTB7A* locus is presented.

DNA genomic sequences of LRF/*ZBTB7A* (1759 bp), *DNMT3A* (871 bp) and *HDAC1* (1489 bp) were synthesized by IDT (Integrated DNA Technologies, Coralville, IA, USA). LRF/*ZBTB7A* and *HDAC1* encompassed the protein coding transcripts NM_015898.4, and NM_004964.3, respectively. To minimize the length of the *DNMT3A* gene and favor its insertion in the vector, only the enzymatic center of the coding sequence was synthesized. The LRF/*ZBTB7A* sequence was ligated into the BspEI restriction site of pEPI-1 episomal expression vector, immediately downstream of the eGFP reporter gene. pEPI-1 includes a functional S/MAR element resulting in successful episomal replication and prolonged retention of the exogenous genes in the nucleus, despite continuous cell mitosis [52]. Before ligation, the linear episomal vector was blunt-ended by the Klenow fragment of DNA polymerase (New England Biolabs GmbH, Frankfurt, Germany), and dephosphorylated with Antarctic Phosphatase (New England Biolabs GmbH, Frankfurt, Germany). After initial transfections of K562 cells with LRF/*ZBTB7A*-eGFP construct, the eGFP (700 bp) was deleted with double digestion (AgeI and BspEI), followed by Klenow treatment to form blunt ends, which were re-ligated with T4 DNA ligase (New England Biolabs GmbH, Frankfurt, Germany). *HDAC1* and the catalytic domain of *DNMT3* were inserted into the episomal vector (with abolished eGFP) as BglII-AseI fragments at relevant sites of the vector. All exogenous genes were flanked by the Kozak consensus sequence (GCCACC) upstream first translation codon and two stop codons at the end of coding sequence (Figure 5A,B). Primer sets utilized in episomal vectors’ construction are listed in Table 1.

### 4.3. LRF/ZBTB7A Knockout Using CRISPR/Cas9 System

The CRISPR (clustered regularly interspaced short palindromic repeats)/Cas9 gene editing technology was used through the homology-directed repair mechanism to knockout the LRF/*ZBTB7A* gene’s expression in K562 cells. Two guide-RNAs (KN222759G1: 5′ AGGATGTCGCTGCTGTGGTC 3′, KN222759G2: 5′ GTGCGACGTGGTGATCCTGG 3′) were designed targeting the second exon of LRF/*ZBTB7A* transcript (NM_015898.4). The donor DNA consisted of a left and a right homologous arm (LHA and RHA) flanking a functional cassette, encoding for the green- fluorescent reporter protein (tGFP) and the puromycin resistance gene (OriGene Technologies, Inc., Rockville, MD, USA) (Figure 5C).

### 4.4. RT-qPCR

Total RNA was extracted from cultivated (untransfected and transfected) K562 clones using the RNeasy Mini kit (74104, Qiagen GmbH, Hilden, Germany) according to the manufacturer’s instructions. 1 μg of total RNA was subjected to cDNA synthesis with the Quantitect Reverse transcription kit (205,311, Qiagen GmbH, Hilden, Germany). Gene’s expression analysis was performed with specific primers sets (Table 1) and qPCR reactions were carried out in ECO Real-Time PCR system (Illumina, CA, USA) using the SsoFast Eva Green SuperMix (Biorad, CA, USA). All reactions were run in triplicates in at least two independent experiments. The 2^−ΔΔCT^ method or Pfaffl equation with PCR efficiency correction was used for data analysis [53]. Fold changes in gene expression were normalized to *GAPDH* reference gene and relative to the calibrator sample (untransfected K562).

### 4.5. Chromatin Immunoprecipitation

ChIP assay was performed using Protein A/G PLUS Agarose (Santa Cruz Biotechnology, Inc., Heidelberg, Germany) according to the Santa Cruz Biotechnology protocol. 2 × 10^7^ cells from studied experimental conditions were cross-linked with 1% formaldehyde. After quenching and cell lysis, chromatin was sonicated with a LABSONIC M sonicator (Sartorius, Göttingen, Germany) to generate 150–300 bp DNA fragments. The polyclonal rabbit anti-ZBTB7A antibody (abcam) was utilized in immunoprecipitation reactions and the rabbit serum IgG fraction (I5006, Sigma-Aldrich Pty Ltd, An affiliate of Merck KGaA, Darmstadt, Germany) as negative control. After reverse crosslinking at 65 °C, immunoprecipitated DNA was recovered/purified via phenol/chloroform extraction and ethanol precipitation. Linear acrylamide (Invitrogen-Thermo Fisher Scientific Inc., Grand Island, NE, USA) was used for pellet visualization and for increasing the precipitation efficiency.

### 4.6. ChIP-Seq Library Construction and Next-Generation Sequencing

Library preparation from ChIP-isolated DNA was performed using the Ion Xpress™ Plus Fragment Kit and the Ion Xpress™ Barcode Adapters 1–16 Kit (both from ThermoFischer Scientific Inc., Grand Island, NE, USA). The protocol for 200 bp read-length libraries and for 50–100 ng input DNA was employed with some modifications. Due to the small amount of input DNA (10 ng), and to avoid adapter concatamerization, 1 μL of P1 adapter and IonXpress™ Barcodes was used instead of 2 μL. Adapter ligation and dual bead size selection for 200 bp read length fragments was performed using AMPure XP beads, followed by separation on magnetic rack, two ethanol washes and a final elution of DNA fragments in 25 μL Low TE. The size selected libraries were then amplified for 16 cycles. The quality was assessed on the Bioanalyzer 2100 (Agilent Technologies, Santa Clara, CA, USA) using a High Sensitivity DNA ChIP (Agilent Technologies, Santa Clara, CA, USA), and quantified via qPCR using the KAPA Library Quantification Kit for Ion Torrent™ [54]. Template preparation and chip loading prior to sequencing was performed on the Ion Chef system using the Ion 540™ Chef kit and an Ion 540™ chip. The loaded chip was then sequenced on an Ion GeneStudio S5 sequencer.

### 4.7. Analysis, Peak Calling and Visualization of ChIP-Seq Data

For downstream analysis, the sequencing data were uploaded to the Galaxy web platform through the usegalaxy.eu public server [55]. The datasets were mapped to hg38 using Bowtie2 with the—very-sensitive-local preset [56]. BigWig files used for visualisation of ChIP-seq data were created using the deeptools bamCoverage function with the following parameters—outFileFormat ‘bigwig’—binSize 1—normalizeUsing RPKM. The initial alignments that include redundant alignments were used to generate the bigWig files, to account for the high degree of sequence duplication within the *HBB* locus that can mask LRF/*ZBTB7A* occupancy. All datasets were visualised in IGV v2.12.2. For peak calling, only the non-redundant alignments from each BAM dataset were used (number of non-redundant alignments >5,652,842) and were compared to the negative (rabbit) IgG control via MACS2 callpeak with the following parameters—gsize ‘2,700,000,000’—*p* value ‘0.05’—bw ‘190’ [57]. The Intersect tool was used to identify peaks located in close proximity to genes (−2000 to +10,000 bps relative to the transcription start site). All raw sequencing data and bigwig files are available at the GEO Repository under the Accession Number GSE200135 (https://www.ncbi.nlm.nih.gov/geo/query/acc.cgi?acc=GSE200135 accessed on 24 May 2022).

### 4.8. Methylation Analysis/Pyrosequencing

DNA methylation was assessed at genomic DNA extracted from K562 cultivated cells using phenol: chloroform: isoamyl acid in 25:24:1 ratio (Sigma-Aldrich Pty Ltd, An affiliate of Merck KGaA, Darmstadt, Germany), according to standard protocols [58]. 1.5 μg of isolated genomic DNA was treated with EpiTech^®^ Bisulfite Kit (QIAGEN GmbH, Hilden, Germany) and bisulfite converted DNA was then used as a template for amplification of regions of interest with PyroMark^®^ PCR Kit (QIAGEN GmbH, Hilden, Germany). PCR products (biotinylated strand) were immobilized with streptavidin-coated sepharose beads (Streptavidin Sepharose™ High Performance, GE HealthCare) and annealed with a Pyrosequencing primer. Reactions were performed in the PyroMark Q24 instrument using PyroMark^®^ Gold Q24 Reagents (QIAGEN GmbH, Hilden, Germany), according to the manufacturer’s instructions. Primer sets are listed at Table 1. Every pyrosequencing reaction was performed in duplicate.

### 4.9. Statistical Analysis

SPSS software version 20.0 was used for data analysis via one-way ANOVA with Bonferroni or Tukey’s multiple comparisons post hoc tests. Results are presented as mean ± SE of at least two biological replicates. Normal distribution of data was assessed by the Shapiro-Wilk test. The *p* values < 0.05 were considered statistically significant.

## Figures and Tables

**Table 1:** Primer sets.

Assay	Gene	Sequence	Primer
**Pyrosequencing CpG assay**	LRF/*ZBTB7A*	GTAGATTTTTTTGTGTTAAGGA	Forward
		AACAAACCCCCAACCTCTAC	5′ biotinylated Reverse
		GGGATTTTTATAGTTTTATTTTTAA	Forward Pyrosequencing
	*HBG2*	5′ ATGGTGGGAGAAGAAAATTAGTT 3′	Forward
		5′ TCTCCTCCAACATCTTCCACATTCA 3′	5′ biotinylated Reverse
		5′ ATAGTTTTAGAGTATTTAGTGA 3′	Forward Pyrosequencing
	*LINE1*	5′ AAGTAAGTTTGGGTAATGG 3′	Forward
		5′ AACAACTCCCATCTACAACTCCC 3′	5′ biotinylated Reverse
		5′ GGGTGGGAGTGAT 3′	Forward Pyrosequencing
**qPCR**	*GAPDH*	5′ AGGGCTGCTTTTAACTCTGGT 3′	Forward
		5′ CCCCACTTGATTTTGGAGGGA 3′	Reverse
	LRF/*ZBTB7A*	5′ GAAGCCCTACGAGTGCAACATC 3′	Forward
		5′ GTGGTTCTTCAGGTCGTAGTTGTG 3′	Reverse
	*HBG2/1*	5′ AACCCCAAAGTCAAGGCACA 3′	Forward
		5′ GATTGCCAAAACGGTCACCA 3′	Reverse
	*HBB*	5′ CTGAGTGAGCTGCACTGTGA 3’	Forward
		5′ ATTGGACAGCAAGAAAGCGA 3′	Reverse
	*DNMT3A*	5′ GGTGCTGTCTCTCTTTGATGGA 3′	Forward
		5′ GGATATGCTTCTGTGTGACGC 3′	Reverse
	*BGLT3*	5′ TCAGGGGTAACACACAAACCA 3′	Forward
		5′ GTCTCATGTGCTGCACGTCT 3′	Reverse
	*HDAC1*	5′ CCCAATGAAGCCTCACCGAA 3′	Forward
		5′ CGGATGGAGCGCAAGAATTT 3′	Reverse
**Cloning**	*LRF/ZBTB7A*	5′ GATGGCCGGCGGCGTGGACG 3′	Forward
		5′ TCATTAGGCGAGTCCGGCTGTGAAG 3′	Reverse
	DNMT3A	5′ GGTAAGATCTGCCACCATGATCCGGGTG 3′	Forward
		5′ CGGCCTATTAATCTACACACACGAAAGT 3′	Reverse
	HDAC1	5′ GGTAAGATCTGCCACCATGGCGCAGAC 3′	Forward
		5′ TAGCCTAGTTATGCTAGCATTAATTCAGG 3′	Reverse

## Data Availability

Not applicable.
